# Characteristics of hospitalized patients with COVID-19 during the first to fifth waves of infection: a report from the Japan COVID-19 Task Force

**DOI:** 10.1186/s12879-022-07927-w

**Published:** 2022-12-12

**Authors:** Ho Lee, Shotaro Chubachi, Ho Namkoong, Takanori Asakura, Hiromu Tanaka, Shiro Otake, Kensuke Nakagawara, Atsuho Morita, Takahiro Fukushima, Mayuko Watase, Tatsuya Kusumoto, Katsunori Masaki, Hirofumi Kamata, Makoto Ishii, Naoki Hasegawa, Norihiro Harada, Tetsuya Ueda, Soichiro Ueda, Takashi Ishiguro, Ken Arimura, Fukuki Saito, Takashi Yoshiyama, Yasushi Nakano, Yoshikazu Mutoh, Yusuke Suzuki, Koji Murakami, Yukinori Okada, Ryuji Koike, Yuko Kitagawa, Akinori Kimura, Seiya Imoto, Satoru Miyano, Seishi Ogawa, Takanori Kanai, Koichi Fukunaga, The Japan COVID-19 Task Force, Ho Lee, Shotaro Chubachi, Ho Namkoong, Takanori Asakura, Hiromu Tanaka, Shiro Otake, Kensuke Nakagawara, Atsuho Morita, Takahiro Fukushima, Mayuko Watase, Tatsuya Kusumoto, Katsunori Masaki, Hirofumi Kamata, Makoto Ishii, Naoki Hasegawa, Norihiro Harada, Tetsuya Ueda, Soichiro Ueda, Takashi Ishiguro, Ken Arimura, Fukuki Saito, Takashi Yoshiyama, Yasushi Nakano, Yoshikazu Mutoh, Yusuke Suzuki, Koji Murakami, Yukinori Okada, Ryuji Koike, Yuko Kitagawa, Akinori Kimura, Seiya Imoto, Satoru Miyano, Seishi Ogawa, Takanori Kanai, Koichi Fukunaga, Kazuhisa Takahashi, Toshio Naito, Makoto Hiki, Yasushi Matsushita, Haruhi Takagi, Ryousuke Aoki, Ai Nakamura, Sonoko Harada, Hitoshi Sasano, Katsunori Masaki, Shinnosuke Ikemura, Satoshi Okamori, Hideki Terai, Takanori Asakura, Junichi Sasaki, Hiroshi Morisaki, Yoshifumi Uwamino, Kosaku Nanki, Yohei Mikami, Sho Uchida, Shunsuke Uno, Rino Ishihara, Yuta Matsubara, Tomoyasu Nishimura, Takunori Ogawa, Toshiro Sato, Masanori Azuma, Ryuichi Saito, Toshikatsu Sado, Yoshimune Miyazaki, Ryuichi Sato, Yuki Haruta, Tadao Nagasaki, Yoshinori Yasui, Yoshinori Hasegawa, Ai Tada, Masayoshi Miyawaki, Masaomi Yamamoto, Eriko Yoshida, Reina Hayashi, Tomoki Nagasaka, Sawako Arai, Yutaro Kaneko, Kana Sasaki, Taisuke Isono, Shun Shibata, Yuma Matsui, Chiaki Hosoda, Kenji Takano, Takashi Nishida, Yoichi Kobayashi, Yotaro Takaku, Noboru Takayanagi, Etsuko Tagaya, Masatoshi Kawana, Yasushi Nakamori, Kazuhisa Yoshiya, Fukuki Saito, Tomoyuki Yoshihara, Daiki Wada, Hiromu Iwamura, Syuji Kanayama, Shuhei Maruyama, Takanori Hasegawa, Kunihiko Takahashi, Tatsuhiko Anzai, Satoshi Ito, Akifumi Endo, Yuji Uchimura, Yasunari Miyazaki, Takayuki Honda, Tomoya Tateishi, Shuji Tohda, Naoya Ichimura, Kazunari Sonobe, Chihiro Tani Sassa, Jun Nakajima, Masumi Ai, Ken Hiroyuki OhtaKokuto, Hideo Ogata, Yoshiaki Tanaka, Kenichi Arakawa, Masafumi Shimoda, Takeshi Osawa, Yukiko Nakajima, Ryusuke Anan, Ryosuke Arai, Yuko Kurihara, Yuko Harada, Kazumi Nishio, Tomonori Sato, Reoto Takei, Satoshi Hagimoto, Yoichiro Noguchi, Yasuhiko Yamano, Hajime Sasano, Sho Ota, Sohei Nakayama, Keita Masuzawa, Tomomi Takano, Kazuhiko Katayama, Koji Murakami, Mitsuhiro Yamada, Hisatoshi Sugiura, Hirohito Sano, Shuichiro Matsumoto, Nozomu Kimura, Yoshinao Ono, Hiroaki Baba, Rie Baba, Daisuke Arai, Takayuki Ogura, Hidenori Takahashi, Shigehiro Hagiwara, Genta Nagao, Shunichiro Konishi, Ichiro Nakachi, Hiroki Tateno, Isano Hase, Shuichi Yoshida, Shoji Suzuki, Miki Kawada, Hirohisa Horinouchi, Fumitake Saito, Keiko Mitamura, Masao Hagihara, Junichi Ochi, Tomoyuki Uchida, Ryuya Edahiro, Yuya Shirai, Kyuto Sonehara, Tatsuhiko Naito, Kenichi Yamamoto, Shinichi Namba, Ken Suzuki, Takayuki Shiroyama, Yuichi Maeda, Takuro Nii, Yoshimi Noda, Takayuki Niitsu, Yuichi Adachi, Takatoshi Enomoto, Saori Amiya, Reina Hara, Noa Sasa, Shuhei Yamada, Toshihiro Kishikawa, Kazunori Tomono, Kazuto Kato Shuhei Yamada, Yuya Ueno, Motoyuki Suzuki, Norihiko Takemoto, Hirotaka Eguchi, Takahito Fukusumi, Takao Imai, Munehisa Fukushima, Masatoshi Takagaki, Haruhiko Kishima, Hidenori Inohara, Haruhiko Hirata, Yoshito Takeda, Atsushi Kumanogoh, Naoki Miyazawa, Yasuhiro Kimura, Reiko Sado, Hideyasu Sugimoto, Akane Kamiya, Naota Kuwahara, Akiko Fujiwara, Tomohiro Matsunaga, Yoko Sato, Takenori Okada, Takashi Inoue, Toshiyuki Hirano, Keigo Kobayashi, Hatsuyo Takaoka, Koichi Nishi, Masaru Nishitsuji, Mayuko Tani, Junya Suzuki, Hiroki Nakatsumi, Hidefumi Koh, Tadashi Manabe, Yohei Funatsu, Fumimaro Ito, Takahiro Fukui, Keisuke Shinozuka, Sumiko Kohashi, Masatoshi Miyazaki, Tomohisa Shoko, Mitsuaki Kojima, Tomohiro Adachi, Motonao Ishikawa, Kenichiro Takahashi, Kazuyoshi Watanabe, Yoshihiro Hirai, Hidetoshi Kawashima, Atsuya Narita, Kazuki Niwa, Yoshiyuki Sekikawa, Hisako Sageshima, Yoshihiko Nakamura, Kota Hoshino, Junichi Maruyama, Hiroyasu Ishikura, Tohru Takata, Takashi Ogura, Hideya Kitamura, Eri Hagiwara, Kota Murohashi, Hiroko Okabayashi, Takao Mochimaru, Shigenari Nukaga, Ryosuke Satomi, Yoshitaka Oyamada, Nobuaki Mori, Tomoya Baba, Yasutaka Fukui, Mitsuru Odate, Shuko Mashimo, Yasushi Makino, Kazuma Yagi, Mizuha Hashiguchi, Junko Kagyo, Tetsuya Shiomi, Kodai Kawamura, Kazuya Ichikado, Kenta Nishiyama, Hiroyuki Muranaka, Kazunori Nakamura, Satoshi Fuke, Hiroshi Saito, Tomoya Tsuchida, Shigeki Fujitani, Mumon Takita, Daiki Morikawa, Toru Yoshida, Takehiro Izumo, Minoru Inomata, Naoyuki Kuse, Nobuyasu Awano, Mari Tone, Akihiro Ito, Toshio Odani, Masaru Amishima, Takeshi Hattori, Yasuo Shichinohe, Takashi Kagaya, Toshiyuki Kita, Kazuhide Ohta, Satoru Sakagami, Kiyoshi Koshida, Morio Nakamura, Koutaro Yokote, Taka-Aki Nakada, Ryuzo Abe, Taku Oshima, Tadanaga Shimada, Kentaro Hayashi, Tetsuo Shimizu, Yutaka Kozu, Hisato Hiranuma, Yasuhiro Gon, Namiki Izumi, Kaoru Nagata, Ken Ueda, Reiko Taki, Satoko Hanada, Naozumi Hashimoto, Keiko Wakahara, Koji Sakamoto, Norihito Omote, Akira Ando, Yu Kusaka, Takehiko Ohba, Susumu Isogai, Aki Ogawa, Takuya Inoue, Nobuhiro Kodama, Yasunari Kaneyama, Shunsuke Maeda, Takashige Kuraki, Takemasa Matsumoto, Masahiro Harada, Takeshi Takahashi, Hiroshi Ono, Toshihiro Sakurai, Takayuki Shibusawa, Yusuke Kawamura, Akiyoshi Nakayama, Hirotaka Matsuo, Yoshifumi Kimizuka, Akihiko Kawana, Tomoya Sano, Chie Watanabe, Ryohei Suematsu, Makoto Masuda, Aya Wakabayashi, Hiroki Watanabe, Suguru Ueda, Masanori Nishikawa, Ayumi Kazuto YoshifujiIto, Saeko Takahashi, Kota Ishioka, Yusuke Chihara, Mayumi Takeuchi, Keisuke Onoi, Jun Shinozuka, Atsushi Sueyoshi, Yoji Nagasaki, Masaki Okamoto, Yoshihisa Tokunaga, Sayoko Ishihara, Masatoshi Shimo, Masafumi Watanabe, Sumito Inoue, Akira Igarashi, Masamichi Sato, Nobuyuki Hizawa, Yoshiaki Inoue, Shigeru Chiba, Kunihiro Yamagata, Hirayasu Kai, Yuji Hiramatsu, Satoru Fukuyama, Keiko Kan-o, Koichiro Matsumoto, Yoshihiro Eriguchi, Akiko Yonekawa, Kensuke Kanaoka, Shoichi Ihara, Kiyoshi Komuta, Koichiro Asano, Tsuyoshi Oguma, Yoko Ito, Satoru Hashimoto, Masaki Yamasaki, Yu Kasamatsu, Yuko Komase, Naoya Hida, Takahiro Tsuburai, Baku Oyama, Yuichiro Kitagawa, Tetsuya Fukuta, Takahito Miyake, Shozo Yoshida, Shinji Ogura, Minoru Takada, Hidenori Kanda, Shinji Abe, Yuta Kono, Yuki Togashi, Hiroyuki Takoi, Ryota Kikuchi, Shinichi Ogawa, Tomouki Ogata, Shoichiro Ishihara, Arihiko Kanehiro, Shinji Ozaki, Yasuko Fuchimoto, Sae Wada, Nobukazu Fujimoto, Kei Nishiyama, Mariko Terashima, Satoru Beppu, Kosuke Yoshida, Osamu Narumoto, Hideaki Nagai, Nobuharu Ooshima, Mitsuru Motegi, Akira Umeda, Kazuya Miyagawa, Hisato Shimada, Mayu Endo, Yoshiyuki Ohira, Hironori Sagara, Akihiko Tanaka, Shin Ohta, Tomoyuki Kimura, Yoko Shibata, Yoshinori Tanino, Takefumi Nikaido, Hiroyuki Minemura, Yuki Sato, Yuichiro Yamada, Takuya Hashino, Masato Shinoki, Hajime Iwagoe, Hiroshi Takahashi, Kazuhiko Fujii, Hiroto Kishi, Tomoo Ishii, Masayuki Kanai, Tomonori Imamura, Tatsuya Yamashita, Masakiyo Yatomi, Toshitaka Maeno, Shinichi Hayashi, Mai Takahashi, Mizuki Kuramochi, Isamu Kamimaki, Yoshiteru Tominaga, Mitsuyoshi Utsugi, Akihiro Ono, Toru Tanaka, Takeru Kashiwada, Kazue Fujita, Yoshinobu Saito, Masahiro Seike, Masahiro Kanai, Ryunosuke Saiki, Yasuhito Nannya, Takayoshi Hyugaji, Eigo Shimizu, Kotoe Katayama, Satoru Miyawaki, Meiko Takahashi, Fumihiko Matsuda, Yosuke Omae, Katsushi Tokunaga, Takafumi Ueno

**Affiliations:** 1 Division of Pulmonary Medicine, Department of Medicine, Keio University School of Medicine, 35 Shinanomachi, Shinjuku-Ku, Tokyo, 160-8582 Japan; 2 Department of Infectious Diseases, Keio University School of Medicine, 35 Shinanomachi, Shinjuku-Ku, Tokyo, 160-8582 Japan; 3 Department of Respiratory Medicine, Juntendo University Faculty of Medicine and Graduate School of Medicine, Tokyo, Japan; 4 Department of Respiratory Medicine, Osaka Saiseikai Nakatsu Hospital, Osaka, Japan; 5 JCHO (Japan Community Health Care Organization) Saitama Medical Center, Internal Medicine, Saitama, Japan; 6 Department of Respiratory Medicine, Saitama Cardiovascular and Respiratory Center, Kumagaya, Japan; 7 Department of Respiratory Medicine, Tokyo Women’s Medical University, Tokyo, Japan; 8 Department of Emergency and Critical Care Medicine, Kansai Medical University General Medical Center, Moriguchi, Japan; 9 Fukujuji Hospital, Kiyose, Japan; 10 Department of Internal Medicine, Kawasaki Municipal Ida Hospital, Kawasaki, Japan; 11 Department of Infectious Diseases, Tosei General Hospital, Seto, Japan; 12 Department of Respiratory Medicine, Kitasato University Kitasato Institute Hospital, Tokyo, Japan; 13 Department of Respiratory Medicine, Tohoku University Graduate School of Medicine, Sendai, Japan; 14 Department of Statistical Genetics, Osaka University Graduate School of Medicine, Suita, Japan; 15 Laboratory for Systems Genetics, RIKEN Center for Integrative Medical Sciences, Kanagawa, Japan; 16 Department of Genome Informatics, Graduate School of Medicine, The University of Tokyo, Tokyo, Japan; 17 Medical Innovation Promotion Center, Tokyo Medical and Dental University, Tokyo, Japan; 18 Department of Surgery, Keio University School of Medicine, Tokyo, Japan; 19 Institute of Research, Tokyo Medical and Dental University, Tokyo, Japan; 20 Division of Health Medical Intelligence, Human Genome Center, The Institute of Medical Science, The University of Tokyo, Tokyo, Japan; 21 M&D Data Science Center, Tokyo Medical and Dental University, Tokyo, Japan; 22 Department of Pathology and Tumor Biology, Kyoto University, Kyoto, Japan; 23 Division of Gastroenterology and Hepatology, Department of Medicine, Keio University School of Medicine, Tokyo, Japan

**Keywords:** COVID-19, Wave of infection, Respiratory infection, Hospitalization

## Abstract

**Background:**

We aimed to elucidate differences in the characteristics of patients with coronavirus disease 2019 (COVID-19) requiring hospitalization in Japan, by COVID-19 waves, from conventional strains to the Delta variant.

**Methods:**

We used secondary data from a database and performed a retrospective cohort study that included 3261 patients aged ≥ 18 years enrolled from 78 hospitals that participated in the Japan COVID-19 Task Force between February 2020 and September 2021.

**Results:**

Patients hospitalized during the second (mean age, 53.2 years [standard deviation {SD}, ± 18.9]) and fifth (mean age, 50.7 years [SD ± 13.9]) COVID-19 waves had a lower mean age than those hospitalized during the other COVID-19 waves. Patients hospitalized during the first COVID-19 wave had a longer hospital stay (mean, 30.3 days [SD ± 21.5], p < 0.0001), and post-hospitalization complications, such as bacterial infections (21.3%, p < 0.0001), were also noticeable. In addition, there was an increase in the use of drugs such as remdesivir/baricitinib/tocilizumab/steroids during the latter COVID-19 waves. In the fifth COVID-19 wave, patients exhibited a greater number of presenting symptoms, and a higher percentage of patients required oxygen therapy at the time of admission. However, the percentage of patients requiring invasive mechanical ventilation was the highest in the first COVID-19 wave and the mortality rate was the highest in the third COVID-19 wave.

**Conclusions:**

We identified differences in clinical characteristics of hospitalized patients with COVID-19 in each COVID-19 wave up to the fifth COVID-19 wave in Japan. The fifth COVID-19 wave was associated with greater disease severity on admission, the third COVID-19 wave had the highest mortality rate, and the first COVID-19 wave had the highest percentage of patients requiring mechanical ventilation.

**Supplementary Information:**

The online version contains supplementary material available at 10.1186/s12879-022-07927-w.

## Background

Coronavirus disease 2019 (COVID-19) was first reported in Wuhan, China, in December 2019, as an outbreak of pneumonia of unknown origin, and with subsequent rapid spread of infection worldwide [1]. The first case of COVID-19 in Japan was identified on January 16, 2020. As of September 2022, the country has experienced five waves of COVID-19, with a cumulative number of approximately 20 million individuals infected with COVID-19 [2]. Epidemics in each country vary in severity according to behavioral restrictions and mitigation strategies [3, 4]. In addition, the COVID-19 situation has become more serious with therapeutic advances, mutant variants, and vaccination [5–7]. Therefore, elucidating characteristics of each COVID-19 wave is important for preparing for future epidemics.

**Fig. 1:**
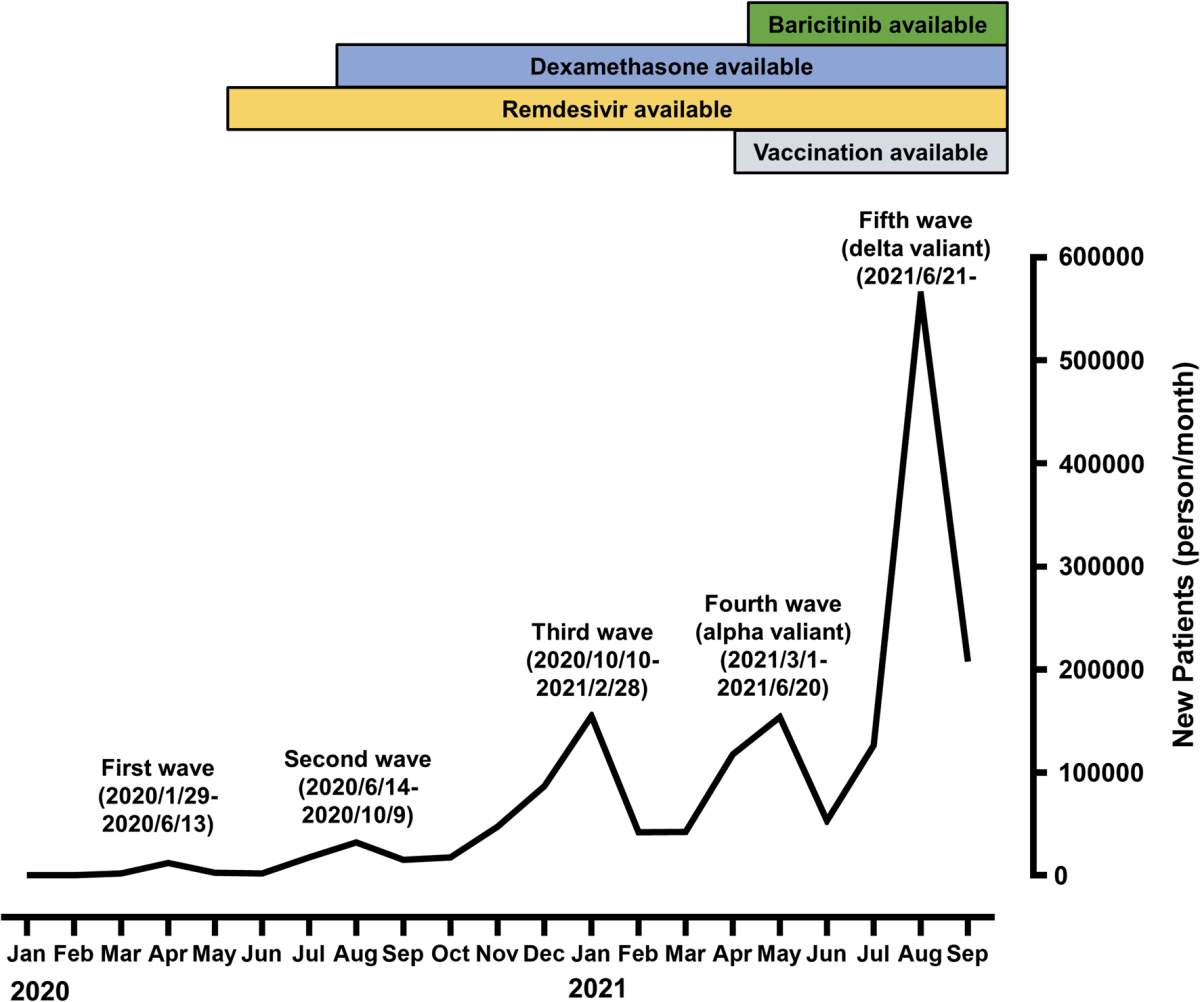
Number of newly diagnosed COVID-19 cases between January 2020 and September 2021 in Japan in each COVID-19 wave. The first case of COVID-19 in Japan was reported on January 16, 2020. Remdesivir was approved by Japan on May 7, 2020. Dexamethasone was first published in the Clinical Practice Guide on July 17, 2020. Baricitinib was approved for use on April 23, 2021

Each COVID-19 outbreak in different regions worldwide has unique characteristics [3, 4, 8]. Similarly, each COVID-19 wave in Japan exhibited unique characteristics. The first COVID-19 wave (2020/1/16 to 2020/6/13), in which the first emergency period was declared, was characterized by a lack of preparation of the health system, with many infections observed in nursing and medical facilities [9, 10]. In the second COVID-19 wave (2020/6/14 to 2020/10/9), after the first emergency was declared, it became apparent that the virus was hiding in the pleasure quarters of large cities and the infection began to spread. In the third COVID-19 wave (2020/10/10 to 2021/2/28), there were more infections than those in the first and second COVID-19 waves as a result of the approach of the Japanese government encouraging economic activities and gradual relaxation of immigration restrictions [11]. In the fourth COVID-19 wave (2021/3/1 to 2021/6/20), the Alpha variant (B.1.1.7), which had a high infectivity and severity rate, became the main strain of the virus [6, 12]. A previous single-center study comparing the fourth COVID-19 wave to the first three COVID-19 waves also showed that the fourth COVID-19 wave was more severe and resulted in a medical crisis in the city [13]. In the fifth COVID-19 wave, the Delta variant (B.1.617), which was associated with increased susceptibility to severe disease, became the main strain, causing a collapse of medical care systems [5, 12]. Figure 1 shows the number of infected people in Japan during each COVID-19 wave [2]. Notably, the country’s medical situation changed dramatically in each COVID-19 wave with advances in treatment and commencement of vaccination [14–17]. Although previous studies have compared each COVID-19 wave [3, 4, 10, 13, 18], the authors could not find any study characterizing Delta waves in Japan. Moreover, there are no comparisons on how the symptoms, severity, and outcomes changed in each COVID-19 wave and according to available therapeutic agents. This study aimed to determine the severity and patient characteristics of each COVID-19 wave using a Japanese nationwide registry to prepare for the possibility of another pandemic in the future.

## Methods

### Study design and settings

The study design and setting have been previously described [19]. This study utilized secondary data from a database. All patients infected with COVID-19 were recruited through the Japan COVID-19 Task Force [20], which was established in February 2020 as a nationwide multicenter consortium in Japan to collect and analyze clinical information of and specimens from patients with COVID-19 at more than 100 facilities nationwide with the aim of overcoming COVID-19. This retrospective cohort study was conducted between February 2020 and September 2021. This study was approved by the Ethics Committee of Keio University School of Medicine (ID: 20200061), and written or oral informed consent was obtained from all participants. The study was performed in accordance with the ethical standards set out in the 1964 Declaration of Helsinki and its later amendments.

### Study population

**Fig. 2:**
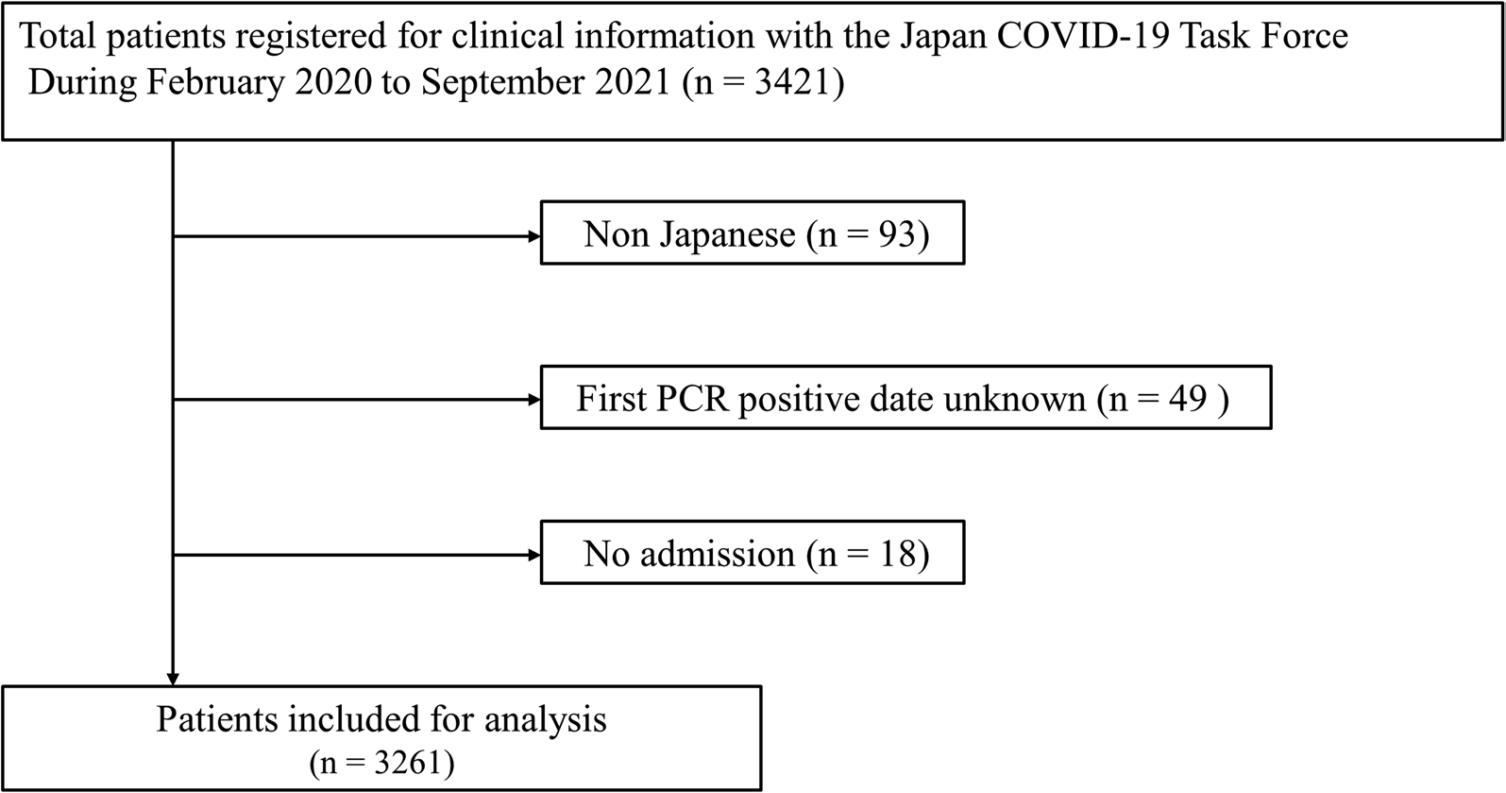
Flowchart describing patient selection. All consecutive patients with COVID-19 aged ≥ 18 years who were hospitalized during the study period (between February 2020 and September 2021) and recruited through the Japan COVID-19 Task Force were included. After excluding 160 patients, 3261 patients were enrolled in this study

Data of consecutive patients aged ≥ 18 years diagnosed with COVID-19 via polymerase chain reaction (PCR) testing or COVID-19 antigen testing at 1 of the 78 participating hospitals in Japan were registered in an electronic case record database by physicians at the affiliated research institution. Participating facilities include a wide range of hospitals in Japan admitting patients with COVID-19. The exclusion criteria were as follows: (1) non-Japanese patients with COVID-19, (2) unknown date of first positive COVID-19 PCR test result, and (3) patients with COVID-19 not hospitalized. Of the 3421 patients who met the inclusion criteria, we excluded 93 non-Japanese patients, 49 patients with unknown first PCR-positive result dates, and 18 patients not hospitalized. Finally, 3261 patients were included in the analysis (Fig. 2).

### Data collection

The following patient data were obtained from electronic case records: first PCR-positive result date, age, sex, body mass index, number of days in the hospital, comorbidities, and clinical symptoms (disturbance of consciousness, fever, malaise, cough/sputum, dyspnea, nasal discharge/pharyngeal pain, gastrointestinal symptoms: abdominal pain/diarrhea/nausea and vomiting, and taste and smell disturbances) during the course of infection. Similarly, we obtained data on laboratory and radiographic findings, complications after hospitalization, medication (ciclesonide, favipiravir, remdesivir, baricitinib, tocilizumab, and steroids), disease severity on admission, and worst condition during hospitalization.

Disease severity was determined considering oxygen requirement: low-flow oxygen therapy, high-flow oxygen therapy including high-flow nasal cannula oxygen therapy (HFNC) or non-invasive positive pressure ventilation, and invasive mechanical ventilation (IMV) [16, 21]. All laboratory and radiographic tests were performed within 48 h of the initial visit or admission according to clinical care needs of patients. A team of clinicians reviewed the collected data. If core data were missing, we contacted the clinician to collect the data. Missing data regarding patient background were assumed to be unknown.

### Statistical analysis

Considering the first PCR-positive result date, the enrolled patients were classified into first–fifth COVID-19 wave groups and clinical characteristics of the patients were compared among the five COVID-19 wave groups. Categorical and continuous variables are presented as number (proportion) and mean ± standard deviation (SD), respectively. Data were compared using the χ^2^ test or Fisher’s exact test for categorical variables, as appropriate, and analysis of variance followed by the Tukey–Kramer method or Kruskal–Wallis test for continuous variables, as appropriate. Statistical significance was set at p < 0.05. Statistical analyses were performed using the JMP 16 program (SAS Institute Japan Ltd., Tokyo, Japan) and R version 4.1.3 (only for Fisher’s exact test). Visualization was performed using GraphPad Prism 9 (GraphPad Software, San Diego, California, USA) and the R package *ggalluvial*.

## Results

### Baseline characteristics of study participants

Baseline patient characteristics are shown in Table 1. The mean age of the 3261 patients with COVID-19 included in this study was 56.9 ± 17.4 years; 67.2% were men. The frequency of each comorbidity ranged from 4.1% to 34.6%. Hypertension (34.6%) and diabetes (21.2%) were the two most prevalent comorbidities.

**Table 1:** Clinical characteristics of Japanese COVID-19 patients

Characteristics	All patients (n = 3261)
Age	56.9 (± 17.4)
Sex, male	2190 (67.2)
BMI	24.8 (± 4.8)
Number of days in the hospital	15.8 (± 1.5)
Comorbidities	
Hypertension	1114 (34.6)
Diabetes	685 (21.2)
Prior cardiovascular disease	329 (10.1)
Cancer	216 (6.7)
COPD	134 (4.1)
Asthma	228 (7.2)
Hyperuricemia	329 (10.2)
Chronic liver disease	136 (4.3)
Chronic kidney disease	227 (7.2)
Smoking, current or former	1412 (47.0)

Data are presented as mean ± SD or n (%)

*BMI* body mass index, *COPD* Chronic obstructive pulmonary disease

### Comparison of characteristics among first to fifth COVID-19 waves

Baseline patient characteristics for each COVID-19 wave are shown in Table 2 and Additional file 5; Table S5. The numbers of patients per COVID-19 wave enrolled in the study were 211, first COVID-19 wave; 837, second COVID-19 wave; 1,197, third COVID-19 wave; 552, fourth COVID-19 wave; and 464, fifth COVID-19 wave. There was a significant difference in the mean age among patients hospitalized during the five COVID-19 waves (p < 0.0001). The mean age was the lowest in the fifth COVID-19 wave. In addition, there was a significant difference in the number of hospitalization days among patients hospitalized during the five COVID-19 waves (p < 0.0001) with the highest number of hospitalization days recorded in the first COVID-19 wave.

**Table 2:** Clinical characteristics of Japanese COVID-19 patients by infection waves

Characteristics	First wave (n = 211)	Second wave (n = 837)	Third wave (n = 1197)	Fourth wave (n = 552)	Fifth wave (n = 464)
Age	57.4 (± 20.3)	53.2 (± 18.9)	61.4 (± 16.4)	57.7 (± 16.4)	50.7 (± 13.9)
Sex, male	111 (52.6)	558 (66.7)	818 (68.3)	371 (67.2)	332 (71.6)
BMI	23.8 (± 5.8)	24.4 (± 4.6)	24.7 (± 4.7)	25.3 (± 5.0)	25.6 (± 4.8)
Number of days in the hospital	30.3 (± 21.5)	13.9 (± 13.1)	16.2 (± 13.8)	15.1 (± 10.8)	13.5 (± 7.8)
Comorbidities					
Hypertension	61 (29.3)	238 (28.9)	483 (41.0)	196 (35.8)	136 (29.4)
Diabetes	40 (19.3)	153 (18.6)	300 (25.3)	112 (20.5)	80 (17.3)
Prior cardiovascular disease	16 (7.8)	78 (9.4)	137 (11.5)	62 (11.3)	36 (7.8)
Cancer	17 (8.1)	41 (5.0)	95 (8.1)	46 (8.4)	18 (3.9)
COPD	9 (4.4)	32 (3.9)	58 (4.9)	27 (5.0)	8 (1.8)
Asthma	19 (9.5)	45 (5.6)	82 (7.0)	46 (8.5)	36 (7.8)
Hyperuricemia	18 (8.8)	73 (8.9)	139 (11.7)	51 (9.3)	48 (10.4)
Chronic liver disease	7 (3.5)	37 (4.7)	37 (3.2)	38 (7.0)	17 (3.7)
Chronic kidney disease	13 (6.9)	45 (5.8)	104 (8.9)	39 (7.3)	26 (5.8)
Smoking, current or former	76 (40.4)	380 (49.3)	496 (45.1)	236 (46.4)	224 (51.3)

Data are presented as mean ± SD or n (%)

*BMI* body mass index, *COPD* Chronic obstructive pulmonary disease

Laboratory data and imaging findings are shown in Additional file 1: Tables S1 and Additional file 2: Table S2, respectively. There were significant differences in laboratory data such as C-reactive protein and D-dimer levels during the five COVID-19 waves (p < 0.0001). Specifically, the C-reactive protein and D-dimer levels were the highest in the first COVID-19 wave compared with other COVID-19 waves.

**Fig. 3:**
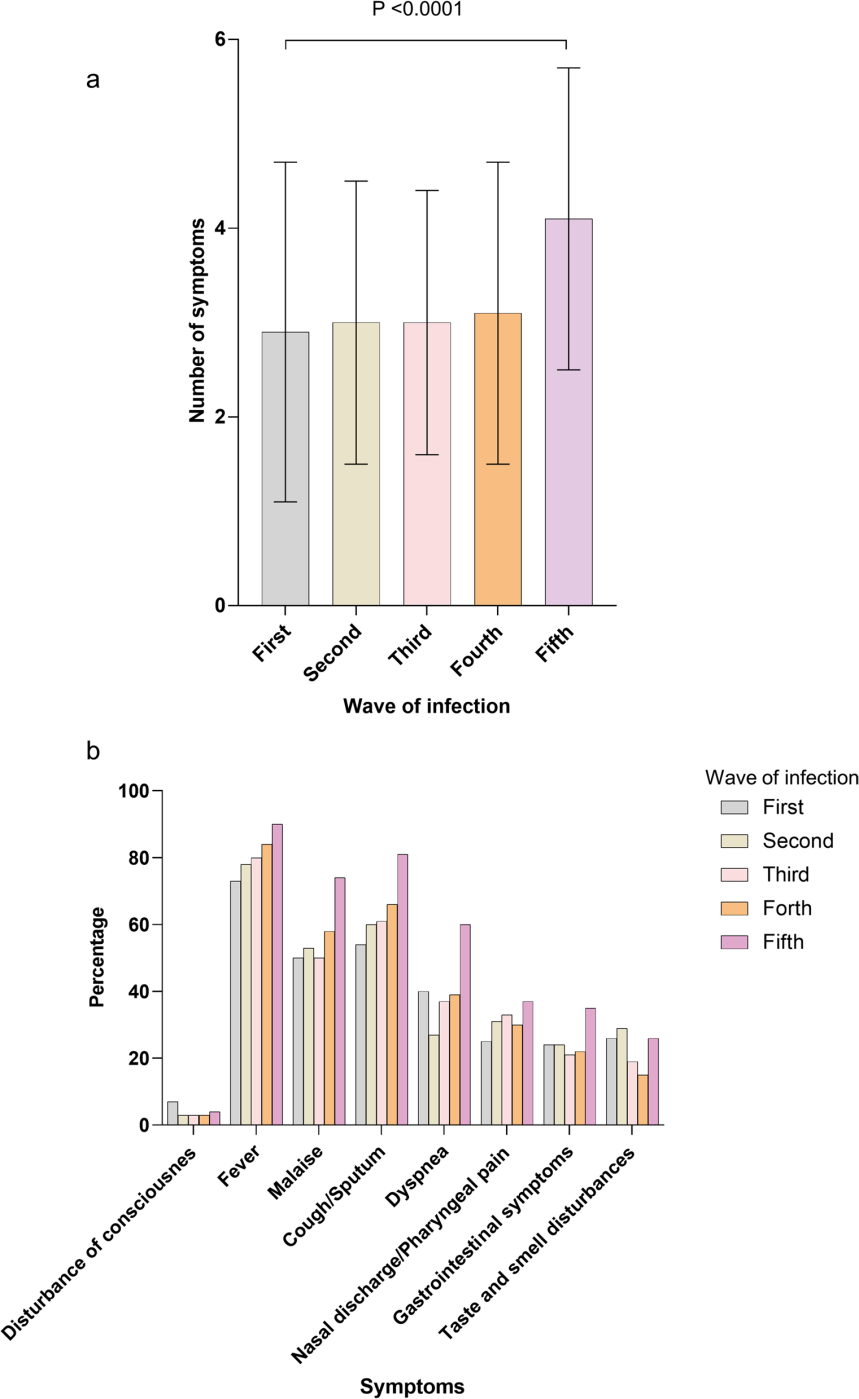
Comparison of clinical symptoms. The total number of eight clinical symptoms per COVID-19 wave of SD is shown **A**. In addition to analysis of variance, all COVID-19 waves were compared using the Tukey–Kramer method (fifth COVID-19 wave vs. first, second, third, and fourth COVID-19 waves: p < 0.0001). A comparison using analysis of variance for each of the eight clinical symptoms (disturbance of consciousness, p = 0.0291; fever, p < 0.0001; malaise, p < 0.0001; cough/sputum, p < 0.0001; dyspnea, p < 0.0001; nasal discharge/pharyngeal pain, p = 0.0256; gastrointestinal symptoms, abdominal pain/diarrhea/nausea and vomiting, p < 0.0001; and taste and smell disturbances, p < 0.0001) are shown in **B**

A comparison of the number of clinical symptoms observed in each COVID-19 wave is shown in Fig. 3A. There was a significant difference in the number of symptoms during the five COVID-19 waves (p < 0.0001). In the fifth COVID-19 wave, patients exhibited more symptoms than those in other COVID-19 waves. There were significant differences in symptoms observed during the five COVID-19 waves, such as fever, malaise, cough/sputum, dyspnea, and gastrointestinal symptoms (p < 0.0001) (Fig. 3B). These symptoms were most frequent in the fifth COVID-19 wave.

### Comparison of medications prescribed per COVID-19 waves

**Fig. 4:**
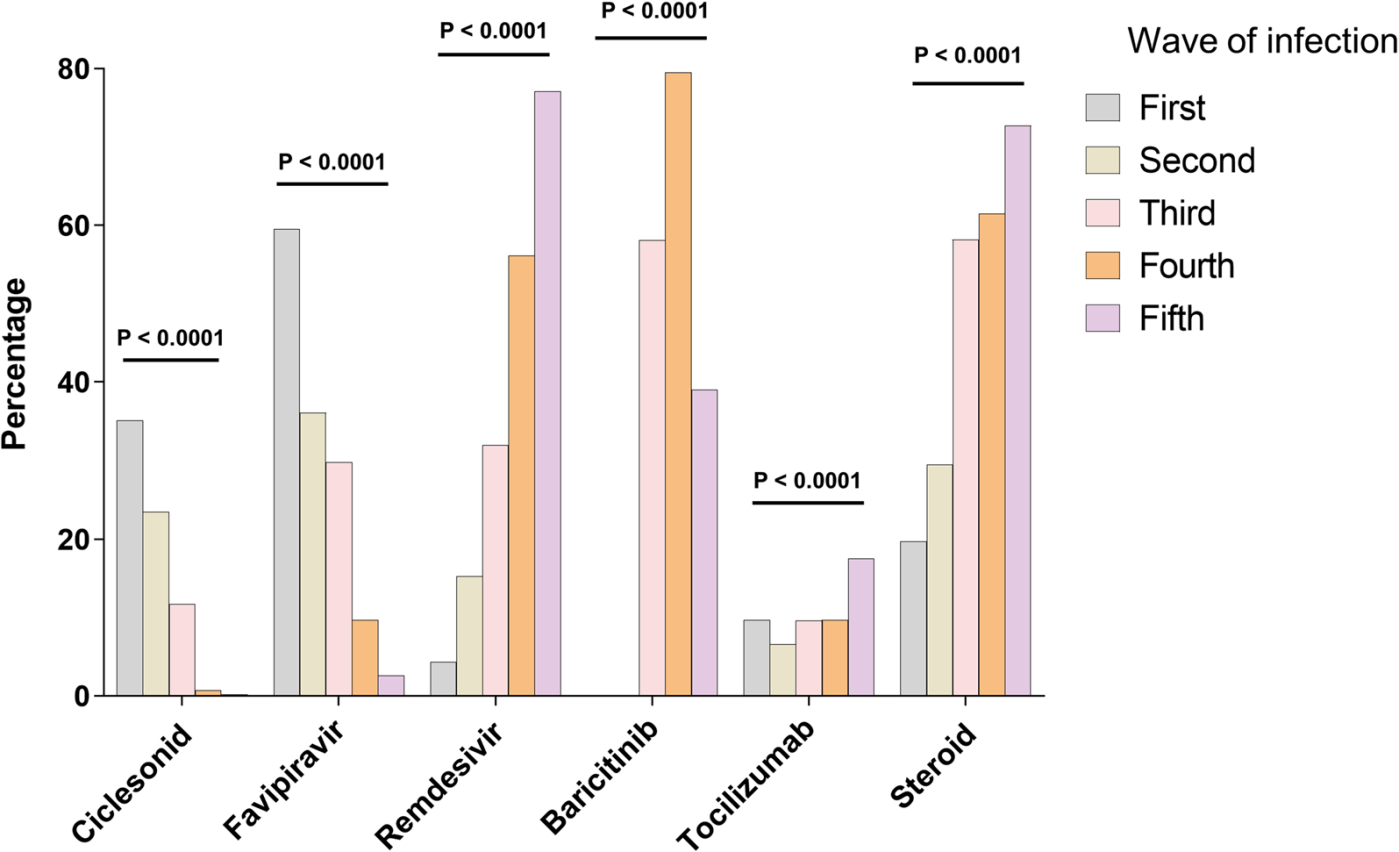
Comparison of medications prescribed per COVID-19 waves. Data are presented as n (%). p values were calculated using the χ^2^ test

A comparison of medications used in each COVID-19 wave is shown in Fig. 4. Significant differences in prescription rates were found among the five waves for all six drugs prescribed for hospitalized patients (p < 0.0001). Ciclesonide and favipiravir were used more frequently in the first COVID-19 wave; however, their frequency of use reduced subsequently. In contrast, remdesivir and steroids were used more frequently in the later COVID-19 waves. Baricitinib was administered from the third COVID-19 wave, and tocilizumab was more commonly used in the fifth COVID-19 wave.

### Comparison of severity of COVID-19

**Fig. 5:**
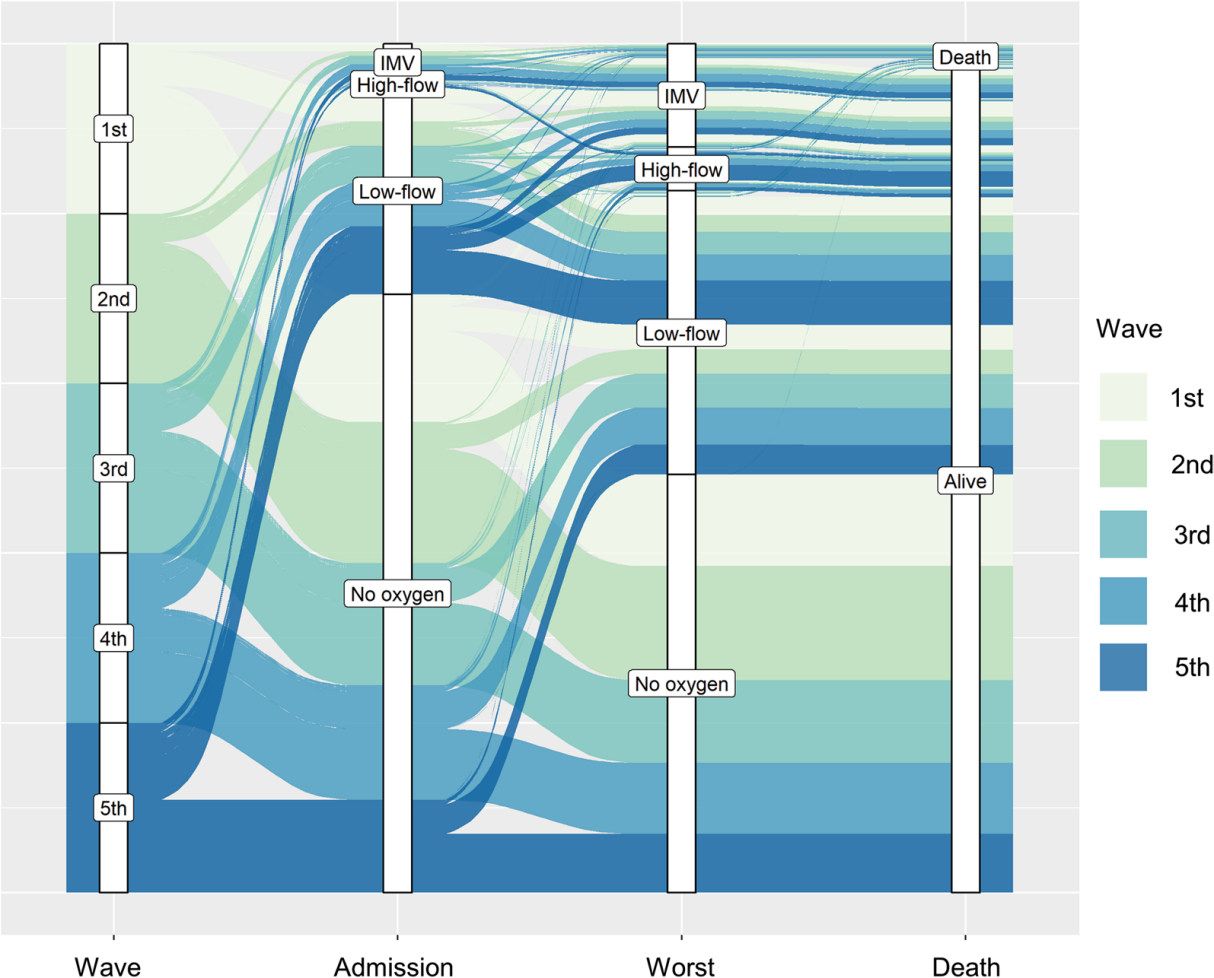
Comparison of COVID-19 severity in each COVID-19 wave. The percentage of COVID-19 severity on admission and the worst condition during hospitalization: low-flow oxygen therapy, high-flow oxygen therapy (using high-flow nasal cannula oxygen therapy or non-invasive positive pressure ventilation) using invasive mechanical ventilation, and hospital death data are shown for each wave. The vertical axis of each wave shows rates of 0–100%

Figure 5 and Additional file 3: Table S3 show the change in severity of the COVID-19 waves at the worst patient condition during hospitalization and at admission. On admission and during the worst in progress, there was a significant difference in the percentage of patients requiring oxygen therapy during the five COVID-19 waves (p < 0.0001), with patients in the fifth COVID-19 wave including the highest percentage of patients requiring oxygen therapy. In addition, during the worst in progress, there was a significant difference in the percentage of patients on high-flow oxygen therapy and IMV during the five COVID-19 waves (p < 0.0001). The fifth COVID-19 wave had the highest percentage of patients on high-flow oxygen therapy, while the first COVID-19 wave had the highest percentage of patients on IMV. In addition, there was a significant difference in the percentage of in-hospital deaths, with the third COVID-19 wave including the highest percentage of in-hospital deaths.

Complications after hospitalization are shown in Table 3 and Additional file 6: Table S6. There was a significant difference among the COVID-19 waves in complications, such as bacterial infection, heart failure, and thrombosis, during the five COVID-19 waves (p < 0.0001), as these were more common in the first COVID-19 wave than in other COVID-19 waves. Patient characteristics of in-hospital deaths were compared for each COVID-19 wave; there were significant differences in diabetes (p = 0.0201) and asthma (p = 0.0059) comorbidity; however, no other significant differences were found (Additional file 4: Table S4, Additional file 5: Table S5, Additional file 6: Table S6).

**Table 3:** Complications after hospitalization by COVID-19 waves

Complications after hospitalization	First wave (n = 211)	Second wave (n = 837)	Third wave (n = 1197)	Fourth wave (n = 552)	Fifth wave (n = 464)
Bacterial infection	44 (21.3)	68 (8.2)	148 (12.6)	54 (9.9)	38 (8.3)
Fungal infection	3 (1.5)	4 (0.5)	17 (1.5)	2 (0.4)	0 (0.0)
Heart failure	15 (7.3)	12 (1.5)	24 (2.1)	5 (0.9)	12 (2.6)
Cardiomyopathy/Myocardial infraction	2 (1.0)	1 (0.1)	9 (0.8)	5 (0.9)	4 (0.9)
Thrombosis	18 (8.7)	7 (0.86)	57 (4.9)	13 (2.4)	5 (1.1)
Liver dysfunction					
Mild	40 (20.7)	162 (20.6)	317 (30.0)	164 (30.2)	167 (36.5)
Moderate	23 (11.9)	65 (8.3)	105 (9.3)	59 (10.9)	81 (17.7)
Severe	14 (7.3)	30 (3.8)	40 (3.5)	17 (3.1)	27 (5.9)
Kidney dysfunction					
Moderate	25 (12.9)	65 (8.3)	165 (14.6)	79 (14.7)	89 (19.6)
Sever	11 (5.7)	18 (2.3)	55 (4.9)	18 (3.4)	20 (4.4)
Hemophagocytic syndrome	7 (3.9)	8 (1.1)	11 (1.1)	6 (1.2)	2 (0.5)

Data are presented as n (%)

## Discussion

In this study, the clinical characteristics of patients with COVID-19 were assessed for different epidemic waves of COVID-19 infection. To our knowledge, this is the first study to compare the detailed clinical characteristics of different COVID-19 waves over a long period of time. It is widely accepted that patient, viral, and social factors contribute in complex ways to the epidemic status of COVID-19 [22, 24]. Particularly, social factors may be diverse from country to country. The Japanese pandemic has been affected by changes in social policies at different time points, such as an increase in the number of people eligible for PCR testing, changes in admission and discharge criteria, and acceptance of patients presenting with mild symptoms of the disease [18]. Therefore, it is postulated that the clinical characteristics and outcomes of patients may differ between COVID-19 waves; however, no previous large-scale multicenter studies have examined this in detail. Consistent with this hypothesis, this study revealed highly distinctive differences in clinical characteristics among the five COVID-19 waves over 1.5 years.

The first COVID-19 wave had the highest percentage of intubated patients during the early stages of infection. The second COVID-19 wave had the largest proportion of young patients and the smallest proportion of patients requiring administration of oxygen. In the third COVID-19 wave, the number of infected patients increased more rapidly than that in the first and second COVID-19 waves, and the percentage of deaths was the highest in this wave. The fourth COVID-19 wave was dominated by the Alpha strain, which was more severe. More patients had required oxygen from the time of admission than that in first–third COVID-19 waves, causing a medical crisis. The fifth COVID-19 wave was dominated by the Delta strain, which was even more severe than the Alpha strain. The number of patients requiring oxygen therapy on admission was the highest during this phase. However, partly owing to advances in treatment and benefits of vaccination, a smaller percentage of patients died or required IMV during the course of admission, and a larger percentage of patients were able to survive up to the point of administration of low-flow or high-flow oxygen therapy [15–17, 21, 25, 26].

Previous studies reported the characteristics of patients with COVID-19 to the fourth COVID-19 wave in one prefecture in Japan [10, 13, 18]. However, the present study investigated differences in disease severity between different COVID-19 waves up to the fifth COVID-19 wave in multiple prefectures in Japan. In the first COVID-19 wave, the rate of the required IMV was higher than that in other COVID-19 waves. There are several possible explanations for this finding. First, there was no established treatment for COVID-19 during the first wave [15, 27, 28]. Second, the high incidence of nosocomial infections may have led to severe conditions in the elderly, with many risk factors [9, 10]. Third, the use of HFNC was avoided because of the risk of causing nosocomial infections by generating aerosols and dispersing the virus [29]. In the fourth and fifth COVID-19 waves, the number of patients requiring oxygen increased because of the effects of variants with a higher rate of severe disease manifestations [5, 6]. Meanwhile, there were fewer cases requiring IMV and fewer deaths in the fourth and fifth COVID-19 waves than in the third COVID-19 wave owing to the accumulation of evidence on the development of treatment methods and vaccination. It is noteworthy that in the fifth COVID-19 wave, which consisted primarily of the more virulent Delta variant, final severity of COVID-19 was reduced despite poor blood test results and severity of illness on admission. This indicates the effectiveness of the COVID-19 treatment and vaccination [15–17]. Furthermore, with each successive COVID-19 wave, the use of HFNC also increased in response to previous reports, and unnecessary IMV use is thought to have decreased as well [15–17, 21, 25, 30].

In this study, the mean age in the fourth COVID-19 wave was lower than that in the third COVID-19 wave [5] [12]. Also, the proportion of patients with severe disease was lower in the fifth COVID-19 wave than in previous COVID-19 waves. These differences may have resulted from the high vaccination rate among the Japanese elderly population. A previous report from Scotland showed that there were fewer severe cases and hospitalizations among elderly patients who had already been vaccinated at least once [31]. Japan implemented a policy of prioritizing vaccination of older adults at high risk of severe disease. The first round of vaccinations of the elderly population began on April 12, 2021, and by the end of July 2021, approximately 80% of older adults had received two doses of a COVID-19 vaccine [14]. This reduced the numbers of patients with serious illness and of hospitalizations among the elderly in the fourth and fifth COVID-19 waves [26].

In this study, ciclesonide and favipiravir were used more frequently in the early COVID-19 waves, while steroids, remdesivir, and baricitinib were used more frequently in the later COVID-19 waves. Currently, COVID-19 treatment is advancing rapidly [30]. Drugs such as ciclesonide and favipiravir, which were experimentally found to be effective, were used in the initial COVID-19 wave of infections [32, 33]. However, their use declined, as validation showed no clear effects [34, 35]. Thereafter, the efficacy of medications such as steroids, remdesivir, and baricitinib was reported [15, 16, 21, 25], and the use of these drugs increased after the third COVID-19 wave. Tocilizumab has also shown therapeutic efficacy [16]; however, its use was not as widespread as that of other drugs, partly because it was not approved for COVID-19 treatment in Japan during the fifth COVID-19 wave. These shifts in treatment are likely to have influenced patient outcomes in the different COVID-19 waves. Moreover, thrombosis and co-infection have been associated with the worst outcomes [36, 37]. Recently, appropriate antibiotic use and prophylactic anticoagulation have been recommended, particularly in severe cases [36, 38, 39]. In this study, there were fewer cases with thrombotic and co-infection complications in the later COVID-19 waves than in the first COVID-19 wave. However, no data were available on whether prophylactic anticoagulation or antimicrobial agents were used appropriately. Nonetheless, appropriate antibiotic use and prophylactic anticoagulation may have reduced these complications in the later COVID-19 waves. Furthermore, the characteristics of patients who died did not differ significantly among the different COVID-19 waves. These results may indicate that the characteristics of patients who died remain constant despite advances in treatment, and these patients may have required further therapeutic intervention. Future studies are warranted to investigate this hypothesis.

The percentage of patients who required oxygen therapy was low in the second COVID-19 wave. This is because PCR testing capabilities had improved compared with that in the first COVID-19 wave, allowing for early diagnosis and treatment. In addition, there was an increase in the number of young people infected, and the elderly, a high-risk group for severe disease, had refrained from leaving their homes [10, 11, 40]. However, in the second COVID-19 wave, no vaccine or treatment was established. In addition, the mortality rate was not significantly different from that in the first COVID-19 wave in the group of patients who required oxygen. From the third COVID-19 wave onward, the number of patients increased more rapidly than that in the first and second COVID-19 waves, and the health system became strained [11]. The third COVID-19 wave had the highest percentage of patients who died without IMV. This may be because the third COVID-19 wave included the highest average aged patients and the largest number of elderly patients, which may have led to a policy of not using IMV even when respiratory failure was severe and ventilator management was necessary.

Our study has several limitations. First, the ratio of the actual number of infected people to the number of patients enrolled in this study was different, and there may have been a selection bias. The largest number of patients was registered in the third COVID-19 wave, but not the fifth COVID-19 wave, which included the largest number of actual infections in Japan. In the fourth and fifth COVID-19 waves, the number of infected patients was high, and a medical crisis occurred, which made the registrars very busy. It is considered that the registration of clinical data did not continue [13]. Second, this study only included hospitalized patients; therefore, it does not reflect the overall picture of COVID-19 in Japan. This applies in particular to time after the third COVID-19 wave when the number of hospitalized patients with mild disease decreased in Japan as a whole. Third, the direct impact of the vaccine on disease severity could not be examined in this study because it was not known whether the patients had been vaccinated. Vaccination has been reported to prevent hospitalization and development of severe symptoms [26]. This may explain differences in outcomes between COVID-19 waves after vaccination became widespread in Japan. Fourth, this study does not consider confounding factors. Previous reports showed that several comorbidities affect the severity of COVID-19. Although viruses and vaccines are confounding factors, the failure to adjust for these confounding factors is a limitation of our study, as a variety of other patient background factors may also affect the severity of COVID-19 [41–45]. Fifth, we could not adjust the data by willingness to accept mechanical ventilation or the decision not to use mechanical ventilation ventilator. Therefore, we may have underestimated the proportion of patients who were severely ill. Further studies are required to confirm these findings.

## Conclusions

We have identified the diverse clinical characteristics of hospitalized patients with COVID-19 in each COVID-19 wave up to the fifth wave in Japan. We found that the characteristics and severity of hospitalized patients with COVID-19 changed with changes in social conditions and use of therapeutic agents in Japan. In the fifth COVID-19 wave in which the Delta variant was the primary source of infection, many patients required oxygenation; however, compared with those in other COVID-19 waves, fewer patients had severe outcomes, such as IMV use or death. These findings may help the Japanese health system respond to future COVID-19 waves.

## Supplementary Information

**Additional file 1. Supplementary Table 1.** Comparison of laboratory parameters.

**Additional file 2. Supplementary Table 2.** Comparison of imaging findings.

**Additional file 3. Supplementary Table 3.** Comparison of severity of COVID-19 patients.

**Additional file 4. Supplementary Table 4.** Comparison of patient characteristics among in-hospital deaths.

**Additional file 5. Supplementary Table 5.** Clinical characteristics of Japanese COVID-19 patients by infection waves with *p* value.

**Additional file 6. Supplementary Table 6.** Complications after hospitalization by COVID-19 waves with *p* value.

## Abbreviations

COVID-19: Coronavirus disease 2019
HFNC: High-flow nasal cannula oxygen therapy
IMV: Invasive mechanical ventilation
